# Screening of Anti‐Atherosclerosis Targets of Ce‐Bai‐Si‐Wei Decoction Based on Multidimensional Data and Study on Cellular Distribution

**DOI:** 10.1155/jdr/9699723

**Published:** 2026-08-05

**Authors:** Wanlu Sheng, Lina Dai, Chengyao Wang, Lun Zhu, Panlei Jia, Xi Yang, Wei Huang, Yuheng Ma, Yingqi Xu

**Affiliations:** ^1^ College of Pharmacy, Inner Mongolia Medical University, Hohhot, China, immu.edu.cn; ^2^ College of Basic Medicine, Inner Mongolia Medical University, Hohhot, China, immu.edu.cn; ^3^ Hohhot Mongolian Medicine of Traditional Chinese Medicine Hospital, Hohhot, China; ^4^ Department of Cell Fate and Diseases, Jilin Provincial Key Laboratory of Women′s Reproductive Health, Jilin Provincial Clinical Research Center for Birth Defect and Rare Disease, The First Hospital of Jilin University, Changchun, China, jlu.edu.cn; ^5^ Key Laboratory of Tropical Translational Medicine of Ministry of Education, College of Basic Medical Sciences, Hainan Medical University, Haikou, China, hainmc.edu.cn; ^6^ College of Bioinformatics Science and Technology, Harbin Medical University, Harbin, China, hrbmu.edu.cn

## Abstract

**Background:**

Atherosclerosis (AS), which causes chronic inflammation, aberrant lipid metabolism and vascular endothelial damage, is the main cause of cardiovascular disease. The Ce‐Bai‐Si‐Wei decoction (CSD) may reduce AS, although its molecular mechanisms are unknown. This study investigates core molecular pathways using multi‐omics integration and experimental validation.

**Method:**

Network pharmacology prediction: Construct a ‘drug‐component–target‐disease’ interaction network using databases like TCMSP, HIT and ETCM to identify active components and targets of CSD. AS regulatory targets and signalling pathways can be identified by integrating microarray data and single‐cell transcriptomics data. In vitro validation: Utilise the OX‐LDL‐induced model of macrophage foam cells to test CSD’s effects on lipid accumulation utilising CCK‐8, Oil Red O and TC/TG quantification. Validate CSD’s regulatory effects on core targets using molecular docking, molecular dynamics simulation, RT‐qPCR and Western blot.

**Results:**

Four core genes, HMOX1, SELE, CD4 and FLT1, were identified as key regulators of inflammation and oxidative stress in AS, and they bind stably to the active components of CSD. Single‐cell data analysis suggested that CSD may improve AS by targeting macrophages and endothelial cells. In vitro, CSD reduced ox‐LDL‐induced lipid accumulation in macrophages and downregulated TNF‐*α* and IL‐1*β*. Mechanistically, the anti‐AS effect of CSD depends on the NRF2/HMOX1 pathway. CSD also downregulated FLT1 and SELE in damaged endothelial cells.

**Conclusion:**

This study investigated the mechanism of CSD in treating AS. CSD acts on the core targets HMOX1, SELE, FLT1 and CD4, with macrophages and endothelial cells as key effector cells. It exerts anti‐AS effects by inhibiting inflammation, reducing lipid accumulation and protecting endothelial function.

## 1. Introduction

Currently, atherosclerosis (AS) is primarily managed with Western pharmacological agents such as statins. However, these drugs are associated with adverse effects, including neuromuscular toxicity [1], highlighting the need for novel therapeutic approaches. Natural medicines have recently gained attention for their potential efficacy and favourable safety profile in AS treatments. Among them, Mongolian medicine has drawn particular interest due to its extensive clinical experience, shaped by the unique lifestyle, dietary habits and environmental conditions of the Mongolian population.

Its foundational ‘three roots and seven elements’ theory proposes that health depends on the balance among Heyi (Qi), Xila (heat) and Badagan (cold/dampness), which, together with seven bodily elements, constitute the physiological and pathological basis of the human body. According to this framework, AS is categorised as a ‘black vein disease’, primarily attributed to excessive heat (Xila) affecting the blood [2–4].

Platycladi Cacumen (ARiCha), Gardeniae Fructus (ZhuRuLa), Chebulae Fructus (ARuLa) and Toosendan Fructus (BaRuLa) are widely used in Mongolian medicine for their lipid‐lowering, anti‐inflammatory and antioxidant properties. Platycladi Cacumen has been shown to reduce serum cholesterol in hyperlipidaemic mice [5]. Gardeniae Fructus contains geniposide, crocins and iridoid glycosides with demonstrated anti‐atherosclerotic activities [6, 7]. Chebulae Fructus exhibits antihyperlipidemic effects [8], while Toosendan Fructus and its constituent toosendanin inhibit adipogenesis and exhibit anti‐inflammatory and antioxidant properties. Although these herbs have shown efficacy in monotherapy for AS, the combined formulation CSD remains unexplored, and its mechanisms require further investigation.

This study systematically elucidated the multi‐target pharmacological mechanisms of CSD using integrated bioinformatics and network pharmacology approaches, overcoming the limitations of conventional methods in studying complex compound formulations. Network pharmacology has emerged as a powerful tool across various disease domains, including cardiovascular [9], neurological [10] and oncological treatments [11]. By incorporating multi‐omics integration, this strategy transcends the constraints of single‐omics analyses, offering a comprehensive perspective on therapeutic interventions through a multi‐dimensional regulatory network of ‘compound–target–pathway–effect’. This research framework not only provides a new avenue for modernising Mongolian medicine but also establishes a theoretical foundation for its innovative development.

## 2. Materials and Methods

### 2.1. Reagents

FBS (cat# C04001‐500), Penicillin–Streptomycin Dual Antibody (cat# C3420‐0100) and DMEM (cat# C3113‐0500) were purchased from ViVaCell (Shanghai, China); Cell Counting Kit‐8 (cat# BS350B) was purchased from Biosharp (Shanghai, China). The Oil Red O staining kit (cat# G1262‐4) was purchased from Servicebio (Beijing, China); TC (cat#A111‐1‐1) and TG (cat#A110‐1‐1) were purchased from Jiancheng Bioengineering Institute (Nanjing, China). Ox‐LDL (cat#YB‐002) was obtained from Yi‐yuan Biotechnologies (Guangzhou, China). Eastep RT Master Mix Kit (cat# Ls2050) was purchased from Promega (Shanghai, China); RNA extraction kit (cat#R0026) and Enhanced BCA Kit (P0010s) was purchased from Beyotime (Shanghai, China). For western blotting, anti‐HMOX1 (cat#10701‐1‐AP), anti‐Nrf2 (cat#16396‐1‐AP), anti‐SELE (cat#20894‐1‐AP) and anti‐FLT1 (cat#13687‐1‐AP) were purchased from Proteintech (Wuhan, China); Anti‐*β*‐actin (cat# LF2015) were purchased from Epizyme (Shanghai, China). HRP‐conjugated Goat Anti‐Rabbit IgG (H + L) (cat# SA00001‐2) and HRP‐conjugated Goat Anti‐Mouse IgG (H + L) (cat#SA0 0001‐2) were purchased from Proteintech (Wuhan, China).

### 2.2. CSD, AS Target Acquisition

Retrieved from the HIT (http://www.badd‐cao.net:2345/), Herb (http://herb.ac.cn/), Symmap (http://www.symmap.org/) and ETCM (http://www.tcmip.cn/ETCM/) databases are CSD targets of four montanas (Platycladi Cacumen, Gardeniae Fructus, Chebulae Fructus and Toosendan Fructus). The TCMSP (https://www.tcmsp-e.com/tcmsp.php) database was screened for active compounds of CSD with OB > 30*%* and DL > 0.18. Disease targets of AS were obtained from the GeneCard database (https://www.genecards.org/). Microarray data related to AS and single‐cell sequencing data were downloaded from the GEO database (https://www.ncbi.nlm.nih.gov/geo/v).

### 2.3. Disease Enrichment Analysis

Disease enrichment analysis of CSD potential targets was performed using the R package ‘clusterProfiler’, encompassing analyses from the Disease Ontology (DO) and DisGeNET databases. A corrected *p* value (*p* adjust) < 0.05 was set as the threshold for significant enrichment, with results visualised using the ‘ggplot2’ package. Input AS targets and CSD targets into the TCMNPAS database, employing the KATZ score and the network distance score to evaluate the relationship between AS and CSD.

### 2.4. Differential Expression Analysis

Differential expression analysis between early and advanced atherosclerotic samples was performed using the R package ‘limma’ on dataset GSE43292, with significance thresholds set at |log₂ FC| > 0.5 and *p* < 0.05. Volcano plots visualizing the results were generated with ‘ggplot2’, annotating the top 10 up‐ and down‐regulated genes. Expression patterns of the top 10 differentially expressed genes (DEGs), ranked by |log₂ FC|, were depicted in a heat map generated using the ‘ComplexHeatmap’ package.

### 2.5. WGCNA Analysis

Weighted gene co‐expression network analysis (WGCNA) was performed using the R package ‘WGCNA’ to identify modules associated with disease progression. The soft‐thresholding power was selected using the pickSoftThreshold function to achieve a scale‐free topology. Gene modules were constructed with the blockwiseModules function, and module eigengenes were calculated via moduleEigengenes. Calculate the Pearson correlation matrices and corresponding *p* values between these gene modules and each phenotype. Finally, select gene modules exhibit similar expression patterns through clustering.

### 2.6. Identify Potential Functional Targets

The DEGs, WGCNA module genes and AS‐related illness targets from the database were intersected and defined as AS disease targets (the AS targets were screened), which were then intersected with CSD targets to identify the prospective targets of CSD against AS. The outcomes were illustrated by constructing a Venn diagram utilising the R package ‘ggvenn’.

### 2.7. Gene Ontology (GO) and Kyoto Encyclopedia of Genes and Genomes (KEGG) Enrichment Analysis

GO and KEGG pathway enrichment analyses were performed using the ‘clusterProfiler’ R package. GO functional categorisation included molecular function (MF), biological process (BP) and cellular component (CC). Enriched terms were ranked by *p* value, and the top 10 entries for each category were visualised using ‘ggplot2’.

### 2.8. Protein–Protein Interaction (PPI) Network Construction

The identified candidate targets were submitted to the STRING database (http://www.string-db.org/) for the creation of a PPI network (interaction score > 0.4). The studies performed in the STRING database were later imported into Cytoscape for display. Essential subnetworks were discerned using MCODE.

### 2.9. Machine Learning Algorithms

To identify critical targets associated with AS, we applied Least Absolute Shrinkage and Selection Operator (LASSO) regression and Support Vector Machine‐Recursive Feature Elimination (SVM‐RFE) algorithms. Using the ‘glmnet’ R package, LASSO regression was performed on the training set to select candidate genes based on the Minimum lambda (*λ*) value. Simultaneously, SVM‐RFE was implemented via the ‘e1071’ package to rank and evaluate feature importance, retaining the feature set with the lowest error rate. The intersection of genes identified by both methods was determined using the ‘ggvenn’ R package, yielding a set of core target genes.

### 2.10. ROC Analysis and Expression Validation

The ROC curve for the core genes was generated using the pROC package in both the training and validation datasets, and the sensitivity and specificity of the core genes were quantitatively assessed based on the AUC value to evaluate their capacity to differentiate between disease and control samples. An AUC value of 0.7 is generally considered to have substantial predictive power. To elucidate the distinctions in important target expression between early and late AS samples, we employed the Wilcoxon test to compare levels in the validation set. Box plots were generated for visualisation utilising the ‘ggplot2’ package. Additionally, we further incorporated an external validation cohort from the Zenodo repository (https://zenodo.org/records/17454209), which includes healthy and atherosclerotic plaque samples. This independent dataset was used to further validate the differential expression and classification performance of the core genes from an independent data source perspective, thereby enhancing the robustness and generalizability of our findings.

### 2.11. Cell Type Annotations

Single‐cell RNA sequencing data (GSE159677) were processed using the Seurat package in R. Initial quality control retained cells with 200–5000 detected genes. The gene expression count per cell exceeds 500, and mitochondrial gene content is below 20%. The data were normalised using the NormaliseData function. The NormaliseData function normalised the filtered data. We selected the top 2000 highly variable genes through variance analysis using FindVariableFeatures. To reduce dimensionality, these highly variable genes were subjected to RunPCA. The Seurat standard process was used to perform tSNE clustering analysis with a clustering resolution of 0.5 to clarify cell clustering affiliation. The CellMarker database and relevant literature were used for cell annotation. We annotated each cell cluster with the marker genes selected by FindAllMarkers. Finally, DotPlot was used to create bubble plots showing marker gene expression across annotated cell types.

### 2.12. Identification of Key Target Activity

The activity levels of principal gene sets in each cell were assessed using the AUCell R package. A threshold value of 0.1082597 was calculated using the target activity scores, subsequently classifying the cells into high‐activity and low‐activity groups. Subsequently, the ratio of high‐activity cells to low‐activity cells across various cell types was quantified and analysed to elucidate the activity distribution of the gene sets within each cell type.

### 2.13. Cellular Communication Analysis

A thorough examination of cell communication was conducted in the high‐ and low‐activity groups using the ‘CellChat’ R package. This approach sought to assess intercellular communication by detecting the expression of known ligand‐receptor pairs both within and among various cell types. The quantity and intensity of intercellular connections were subsequently compared between the two groups to investigate disparities in cellular communication strength.

### 2.14. Molecular Docking

The crystalline structures of the target proteins, including HMOX1 (PDB ID: 1N45), SELE (PDB ID: 1G1T), FLT1 (PDB ID: 1RV6) and CD4 (PDB ID: 2NY1), were individually downloaded via the PDB database (https://www.rcsb.org/). In Discovery Studio, the Clean Protein module is used to remove non‐standard amino acids and other heteroatoms and add hydrogen atoms to the protein. The protein is then further preprocessed using the Prepare Protein module. Small molecules are preprocessed through the ‘Prepare Ligands’ module. The ‘From Current Selection’ module is then used to determine the active site or binding pocket of each target protein. The ‘Dock Ligand (CDOCKER)’ module then performs precise molecular docking to dock small molecules to proteins, and ‘‐CDOCKER_ENERGY’ is calculated to evaluate the binding capacity.

### 2.15. Molecular Dynamics Simulations

The stability of the complexes was evaluated by molecular dynamics simulations using CASB‐flex (https://bitbucket.org/lcbio/cabsflex). Default values were selected for all parameters.

### 2.16. CSD Preparation

All herbs were obtained from Hohhot Meng Medicine Hospital of Traditional Chinese Medicine. Preparation of CSD: The four Mongolian herbs in the prescription were pulverised into coarse powder and mixed in a 1:1:1:1 ratio, combined with 500 mL of water and heated under reflux for 1.5 h. After filtration, the filtrate was concentrated using a rotary evaporator to a final volume of 75 mL.

### 2.17. Animals and Acquisition of Medicated Serum

Twelve healthy male Wistar rats (250–290 g) [permit no. SYXK(MONG) 2020‐0003] were provided by Beijing Viton Lever Laboratory Animal Technology Co. Ltd. After acclimatisation for 7 days, the rats received 1.35 g/kg CSD via oral gavage twice daily for 7 days. Blood was collected from the abdominal aorta 1.5 h after the final administration under urethane anaesthesia. Serum was separated by centrifugation at 860 g/min for 15 min, inactivated at 56°C for 30 min, filter‐sterilised (0.22 *μ*m) and stored at −80°C for subsequent use.

### 2.18. Cell Culture

The RAW264.7 murine macrophage cell line was grown in DMEM medium (10% heat‐inactivated FBS, 1% penicillin/streptomycin) in a 5% CO₂ incubator at 37°C. Cells in the logarithmic growth phase were used for subsequent tests.

The HUVECS cell line was grown in ECM medium (10% heat‐inactivated FBS, 1% penicillin/streptomycin) in a 5% CO₂ incubator at 37°C. Cells in the logarithmic growth phase were used for subsequent tests.

### 2.19. CCK‐8

RAW264.7 cells were grown in 96‐well plates for study and treated with CSD‐mediated serum at concentrations of 2%, 4%, 6%, 8% and 10% for 24 h. The medium was subsequently replaced with a 10% CCK‐8 solution, and the cells were incubated for 1 h. Absorbance measurements were obtained at a wavelength of 450 nm.

### 2.20. Oil Red O Staining

Cells were washed twice with PBS, fixed for 30 min and rinsed twice with distilled water. They were then treated with 60% isopropanol for 20–30 s and stained with Oil Red O solution for 1 h. After rinsing in 60% isopropanol for 10–20 s to clear the background, the cells were washed 2–5 times with distilled water. Nuclei were counterstained with Mayer’s Haematoxylin for 1–2 min, followed by two distilled water rinses. Bluing was performed for 1 min, and samples were mounted in water for microscopic examination.

### 2.21. Determination of TC and TG Content

The cell suspension was collected and centrifuged to obtain the cell pellet. The cell pellet was resuspended in PBS and homogenised by sonication, followed by the addition of standards, samples, and working solutions as per the instructions. Agitate the well plate thoroughly, incubate at 37°C for 10 min and thereafter, measure the absorbance of each well at 500 nm.

### 2.22. Quantitative Real‐Time PCR (RT‐qPCR) Analysis

Total RNA was extracted using the RNeasy Animal RNA Isolation Kit, followed by reverse transcription with the Eastep RT Master Mix. Real‐time PCR was performed on an iCycler iQ system with SYBR Premix Ex Taq II. *β*‐Actin was used as a reference gene for normalisation. Primer sequences are provided in Table 1.

**Table 1:** The sequence for primers for PCR.

Gene	Forward primer (5 ^′^−3 ^′^)	Reverse primer (5 ^′^ −3 ^′^)
HMOX1	TCCATGTTGACTGACCACGACT	CCCACCCCTCAAAAGATAGCC
Nrf2	AAAATCATTAACCTCCCTGTTGAT	CGGCGACTTTATTCTTACCTCTC
TNF‐*α*	GGTGCCTATGTCTCAGCCTCTT	GCCATAGAACTGATGAGAGGGAG
IL‐1*β*	TGGACCTTCCAGGATGAGGACA	GTTCATCTCGGAGCCTGTAGTG
*β*‐actin	AAGACCTCTATGCCAACAC	CTGCTTGCTGATCCACA

### 2.23. Western Blot (WB) Analysis

Protein extracts were resolved by SDS‐PAGE on 10% or 12% gels and transferred to methanol‐activated PVDF membranes. After blocking with rapid blocking buffer for 20 min at room temperature, the membranes were incubated with primary antibody overnight at 4°C. Following TBST washes, membranes were probed with secondary antibody for 1 h at room temperature. The protein bands were visualised using chemiluminescence.

### 2.24. Statistical Analysis

The experimental results are presented as mean ± standard deviation (SD). Differences between groups were analysed using one‐way ANOVA via GraphPad Prism 9.5 software, with a significance level set at *p* < 0.05 to determine statistical significance.

## 3. Results

### 3.1. Analysis of the Relationship Between CSD and AS

Twenty‐five bioactive compounds from CSD were identified using the TCMSP database, as shown in Table 2. A total of 854 CSD‐related targets were obtained by integrating data from the HIT, ETCM, Herb and Symmap databases.

**Table 2:** Active ingredients in CSD.

Compound	Molecular weight (MW)	Drug likeness (DL)	Oral bioavailability (OB)
Hinokinin	354.38	56.5	0.64
DNOP	390.62	40.59	0.4
Isopimaric acid	302.5	36.2	0.28
Beta‐sitosterol	414.79	36.91	0.75
Kaempferol	286.25	41.88	0.24
Quercetin	302.25	46.43	0.28
Ellagic acid	302.2	43.06	0.43
7‐Dehydrosigmasterol	414.79	37.42	0.75
Chebulic acid	356.26	72	0.32
Ellipticine	246.33	30.82	0.28
Peraksine	310.43	82.58	0.78
Cheilanthifoline	325.39	46.51	0.72
Mandenol	308.56	42	0.19
Ethyl linolenate	306.54	46.1	0.2
Stigmasterol	440.83	43.41	0.76
MEDIORESINOL	388.45	57.2	0.62
Crocetin	328.44	35.3	0.26
Ammidin	270.3	34.55	0.22
Isoimperatorin	270.3	45.46	0.23
Ethyl oleate (NF)	310.58	32.4	0.19
3‐Methylkempferol	300.28	60.16	0.26
Deoxypicropophyllotoxin	398.44	52.7	0.83
(R)‐(6‐Methoxyquinolin‐4‐yl)((1R,2R,4R,5S)‐5‐vinylquinuclidin‐2‐yl)methanol	324.46	55.88	0.4
(+)‐Balanophonin	356.4	54.74	0.4
Corymbosin	358.37	51.96	0.41

**Figure 1:**
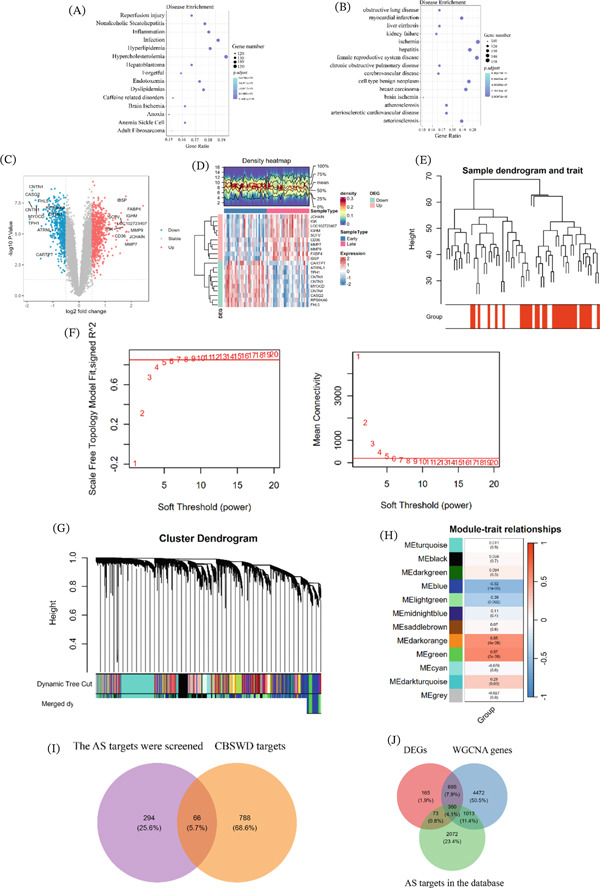
Analysis of the association between CSD and AS and preliminary screening of CSD targets for AS treatment. (A) Disease ontology (DO) enrichment analysis of CSD targets. (B) DisGeNET disease enrichment analysis. (C) Volcano plot of differentially expressed genes (DEGs) between early and late AS stages. (D) Heatmap of DEGs. (E) Sample clustering dendrogram. (F) Selection of soft‐thresholding power (*β*) for scale‐free co‐expression network construction. (G) Identification of gene modules through hierarchical clustering. (H) Module–trait correlation heat map. (I) Intersection of AS‐associated targets from DEGs, WGCNA modules and disease databases. (J) Overlap between CSD and AS target genes.

Functional enrichment analysis using DO and DisGeNET databases revealed significant associations between CSD targets and hyperlipidaemia, hypercholesterolaemia, dyslipidaemia, inflammation, AS and vascular disorders (Figure 1A,B). Further analysis using the TCMNPAS database also showed a significant correlation between CSD and AS, supported by high KATZ and Network Distance scores (Table 3). These results suggest CSD may have therapeutic potential against AS and merit further investigation.

**Table 3:** Network correlation scores for CSD and AS.

Analysis method	Scoring metrics	Score
KATZ	Overlap score	531
	Path 1 score	44.157
	Path 2 score	12.044
	Path 3 score	3.916
	Total score	591.117
	Random score median	158.705
	*p* value	6.343 × 10^−84^
Network distance	Mean distance	2.489
	Random score median	2.634
	*p* value	0.084

### 3.2. Preliminary Screening of CSD Targets for AS

**Figure 2:**
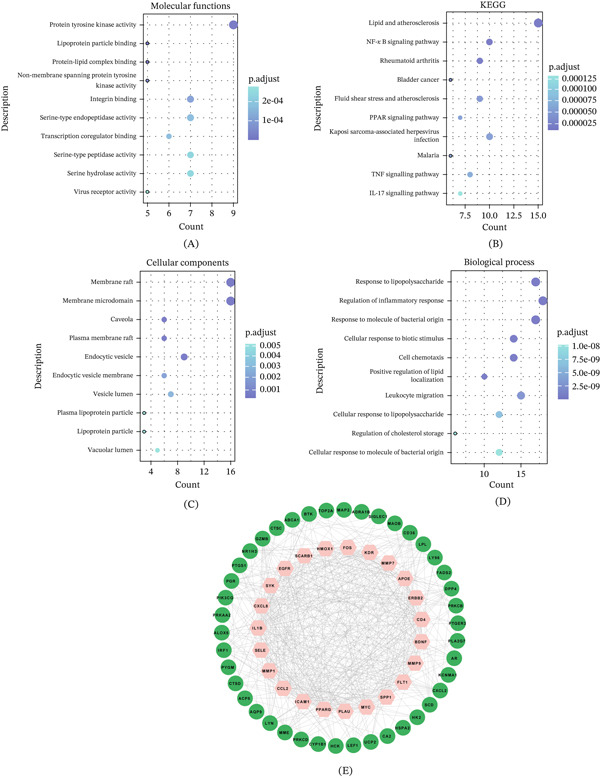
Functional enrichment of potential CSD‐AS targets and PPI network screening. (A) Biological processes; (B) cellular components; (C) molecular functions; (D) pathway enrichment; (E) protein–protein interaction (PPI) network construction.

To identify therapeutic targets of CSD against AS, we analysed the GSE43292 dataset using the ‘limma’ R package. DEGs between early and late AS stages were identified (|log₂FC| > 0.5, *p* < 0.05) and visualised via volcano plot and heat map (Figure 1C,D). WGCNA was performed after quality control and outlier removal (Figure 1E). A scale‐free co‐expression network was constructed with soft threshold *β* = 7 (*R* ^2^ = 0.85) (Figure 1F). Cluster the targets into several modules and label them with different colours (Figure 1G), yielding 11 distinct gene modules. The green (cor = 0.57, *p* = 2e^−06^) and blue (cor = −0.52, p = 1e^−05^) modules showed the strongest correlation with AS progression (Figure 1H). Integration of DEGs, key module genes, and AS in the databases identified 360 potential AS targets (Figure 1I). Intersection with CSD targets revealed 66 candidate therapeutic targets (Figure 1J). Functional enrichment analysis highlighted involvement in inflammatory regulation, leukocyte migration, integrin binding, lipid metabolism, AS and NF‐*κ*B signalling pathways (Figure 2A–D).

### 3.3. Optimisation of CSD as an Anti‐AS Target

**Figure 3:**
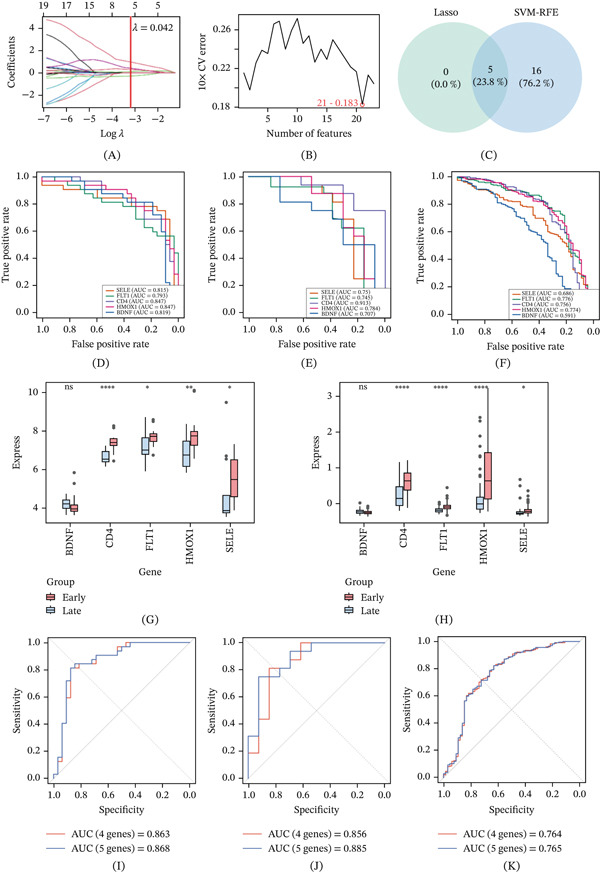
Optimization of CSD targets for AS and validation of the diagnostic performance of key targets. (A) LASSO regression gene coefficient curve; (B) cross‐validation error rate curve analysed for SVM‐RFE; (C) the intersection of feature genes from LASSO and SVM‐RFE algorithms was used; (D–F) ROC curves of individual genes based on the training set GSE43292 (D), test set GSE28829 (E), and the external validation cohort (F); (G–H) expression differences of five key target genes between early‐ and late‐stage samples in the internal dataset (G) and external validation cohort (H); (I–K) comparison of fitted ROC curves constructed using four‐gene and five‐gene models in the training set GSE43292 (I), test set GSE28829 (J), and external validation cohort (K).

A PPI network was constructed (interaction score > 0.4), and MCODE analysis identified a key functional subnetwork comprising 23 core targets (Figure 2E). To elucidate the core mechanisms of CSD in AS pathogenesis, we refined the 23 candidate targets to a core set using machine learning feature selection via LASSO regression and SVM‐RFE. LASSO regression, with an optimal lambda.min value of 0.042, selected a subset of feature genes (Figure 3A). SVM‐RFE was then applied to rank genes by importance, with the feature set yielding the lowest error rate identified as optimal (Figure 3B). The intersection of genes identified by both methods yielded five core targets: CD4, HMOX1, FLT1, SELE and BDNF (Figure 3C).

### 3.4. Validation of the Diagnostic Performance of Key Targets

The diagnostic value of these targets was evaluated using ROC curve analysis. In the training, testing and external validation cohorts, all candidate targets achieved AUC values greater than 0.7 in ROC curve analyses (Figure 3D–F), indicating their favourable ability to discriminate between disease and control samples. Gene expression analysis further confirmed that CD4, HMOX1, FLT1 and SELE consistently exhibited significant differential expression across multiple datasets (*p* < 0.05; Figure 3G), with consistent results also observed in the external validation cohort (Figure 3H), further supporting their potential critical roles in the development and progression of AS. In addition, we constructed a combined ROC model based on the candidate genes to evaluate its overall diagnostic performance. The results showed that the integrated model demonstrated good diagnostic accuracy across all three datasets, and the removal of BDNF did not lead to a noticeable decrease in AUC (Figure 3I–K), suggesting that BDNF contributed limited predictive value to the model. Therefore, CD4, HMOX1, FLT1 and SELE were ultimately retained as the core genes for subsequent analyses.

### 3.5. Validation of Key Targets Based on Molecular Docking

**Figure 4:**
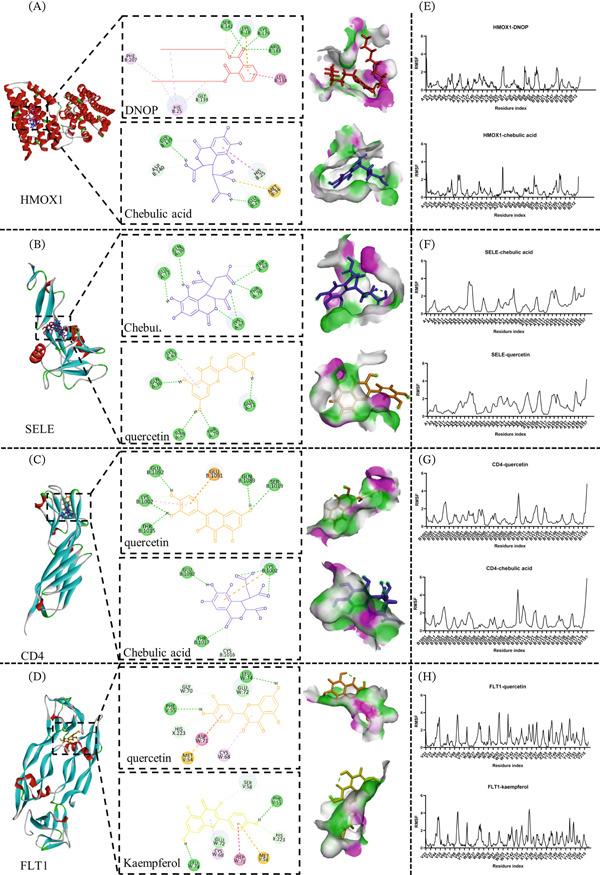
Validation of key targets based on molecular docking. (A) Binding of HMOX1 to DNOP and chebulic acid. (B) Docking of SELE with chebulic acid and quercetin. (C) Interaction of CD4 with quercetin and chebulic acid. (D) Binding of FLT1 to quercetin and kaempferol. For each panel, from left to right: overall binding pose, 2D interaction diagram, and binding pocket with H‐bond acceptors/donors. (E) The RMSF values of HMOX1 docking complexes with DNOP and chebulic acid fluctuated. (F) The RMSF values of SELE docking complexes with quercetin and chebulic acid fluctuated. (G) The RMSF values of CD4 docking complexes with quercetin and chebulic acid fluctuated. (H) The RMSF values of CD4 docking complexes with quercetin and kaempferol fluctuated.

To identify key active constituents in CSD against AS, molecular docking was performed between its chemical components and the four core targets. The top two compounds per target, selected based on docking scores, are listed in Table 4. HMOX1 showed the strongest binding with DNOP and chebulic acid (Figure 4A), SELE with chebulic acid and quercetin (Figure 4B), CD4 with chebulic acid and quercetin (Figure 4C) and FLT1 with quercetin and kaempferol (Figure 4D). Among all targets, HMOX1 exhibited the highest overall affinity with CSD components. Chebulic acid and quercetin demonstrated superior binding to multiple targets, suggesting their pivotal role in the anti‐AS effects of CSD. We used CABS‐FLEX 2.0 to evaluate the root mean square fluctuation (RMSF) of the top protein–ligand complexes. The RMSF value calculates the fluctuations of each amino acid residue as it interacts with its ligand throughout the simulation. Higher RMSF values indicate more flexible residues, whereas lower RMSF values indicate restricted movement. For all studied complexes, we observed small changes in RMSF for all complexes. Active pocket amino acid residues that interact with ligands during docking remain highly stable (Figure 4E–L).Based on these results, we further investigated the role of CSD in regulating HMOX1 and its pathways in AS.

**Table 4:** Interactions between CSD small molecules and key targets.

Gene	Compound	‐CDOCKER_ENERGY	Mongolian medicine
HMOX1	DNOP	45.6791	Platycladi Cacumen
	Chebulic acid	41.4155	Chebulae Fructus
SELE	Chebulic acid	39.0754	Chebulae Fructus
	Quercetin	23.2106	Platycladi Cacumen; Gardeniae Fructus; Toosendan Fructus
CD4	Quercetin	32.3735	Platycladi Cacumen; Gardeniae Fructus; Toosendan Fructus
	Chebulic acid	38.8166	Chebulae Fructus
FLT1	Quercetin	28.777	Platycladi Cacumen; Gardeniae Fructus; Toosendan Fructus
	Kaempferol	23.8054	Platycladi Cacumen; Gardeniae Fructus

### 3.6. Analysis of CSD‐Mediated Cell Subpopulations Based on Single‐Cell Data

**Figure 5:**
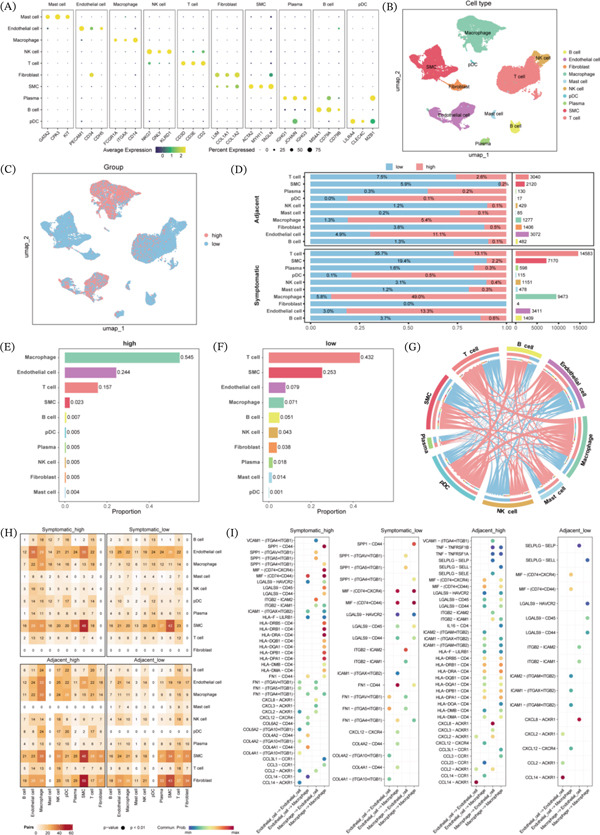
Analysis of CSD‐mediated cell subpopulations based on single‐cell data. (A) Dot plot displaying canonical marker gene expression; (B) cell type annotation of 10 distinct populations; (C) separation of cells based on activity threshold into high‐ and low‐activity groups; (D) proportions of high‐ and low‐activity cells across different disease groups and cell types; (E, F) composition of high‐activity (E) and low‐activity (F) groups per cell type; (G–H) intercellular interaction strength (G) and interaction counts (H) among different cell types in the high‐score and low‐score groups; (I) composition of signalling pathways and their corresponding interaction strengths between macrophages and endothelial cells across the four groups.

After quality control and preprocessing of the GSE159677 single‐cell RNA sequencing dataset, 2000 highly variable genes were identified and subjected to principal component analysis (PCA) for dimensionality reduction. Cell clustering was performed using a resolution parameter of 1.5, resulting in 35 distinct cell clusters. Cell type annotation was systematically conducted by integrating differential expression results, the CellMarker database, and previously published literature. The expression patterns of canonical marker genes were visualized using dot plots (Figure 5A), and a total of 10 major cell types were ultimately identified (Figure 5B).

To evaluate the activity of the four core targets at single‐cell resolution, cells were stratified into high‐ and low‐activity groups based on the 70th percentile threshold of the four‐gene signature score (Figure 5C). Notably, macrophages and endothelial cells were significantly enriched in the high‐activity group, particularly in samples derived from the core regions of atherosclerotic plaques (advanced lesions) (Figure 5D–F), suggesting that these two cell types may represent key effector populations of the AS‐associated cellular state (CSD).

Cell–cell communication analysis revealed that overall intercellular interaction activity was markedly higher in the high‐score groups compared with the low‐score groups. Specifically, macrophages and endothelial cells exhibited the strongest intercellular communication in the Symptomatic_high group, whereas vascular smooth muscle cells (SMCs) showed more active communication patterns in the Adjacent_high group. In contrast, all low‐score groups displayed substantially attenuated intercellular communication. Further analysis of signalling pathways between macrophages and endothelial cells demonstrated that, compared with other groups, the advanced‐lesion high‐score group exhibited significantly enhanced MHC‐II, SPP1 and MIF signalling pathways. These findings suggest that high expression of the four key genes may promote immune regulation‐ and inflammation‐related interactions between macrophages and endothelial cells, thereby contributing to the initiation and progression of advanced atherosclerotic lesions. Collectively, these results further highlight the critical role of macrophages and endothelial cells in mediating the anti‐atherosclerotic effects of CSD.

### 3.7. A Study on the Effects and Mechanisms of CSD in Models of Macrophage Foam Cell Formation and Endothelial Cell Damage

**Figure 6:**
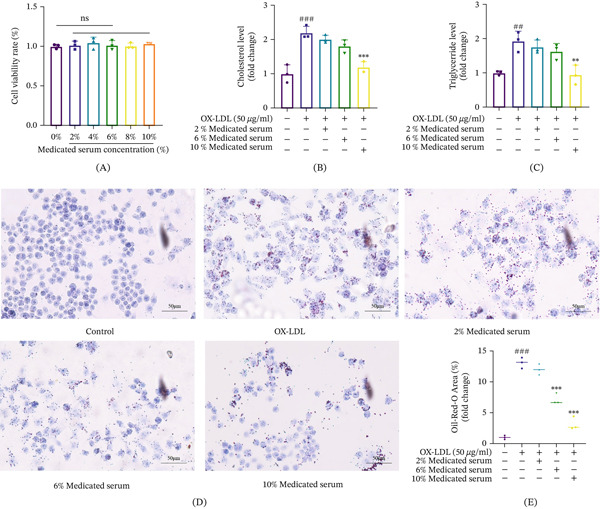
The effect of CSD medicated serum on lipid accumulation. (A) Cell viability was assessed by the CCK‐8 assay following 24‐h treatment with 2%–10% CSD‐medicated serum. (B, C) Levels of total cholesterol (TC) and triglycerides (TG) in ox‐LDL‐induced macrophages were determined after CSD serum treatment. (D) Lipid accumulation was detected by Oil Red O staining (40× magnification; scale bar: 50 *μ*m). (E) Quantitative analysis using oil red O staining. (Data are presented as mean ± SD. #*p* < 0.05, ##*p* < 0.005, ###*p* < 0.001 compared with the control group;  ^∗^ *p* < 0.05,  ^∗∗^ *p* < 0.005,  ^∗∗∗^ *p* < 0.001 compared with the ox‐LDL group.)

To evaluate the potential anti‐atherosclerotic effects of CSD, we established an in vitro model using macrophages, which single‐cell analysis indicated were enriched in high‐activity groups. CCK‐8 assays confirmed (2%, 4%, 6%, 8%, 10%) that CSD‐medicated serum exhibited no significant cytotoxicity at any tested concentration (Figure 6A), demonstrating favourable biocompatibility. Based on prior findings, RAW264.7 cells were induced with 50 *μ*g/mL ox‐LDL to simulate foam cell formation. Lipid metabolism analyses revealed that 10% CSD‐medicated serum significantly reduced intracellular total cholesterol (TC) and triglyceride (TG) levels (Figure 6B,C). Oil Red O staining further confirmed a marked decrease in lipid accumulation, with 10% serum showing the strongest effect (Figure 6D,E). Consequently, this concentration was selected for subsequent experiments.

**Figure 7:**
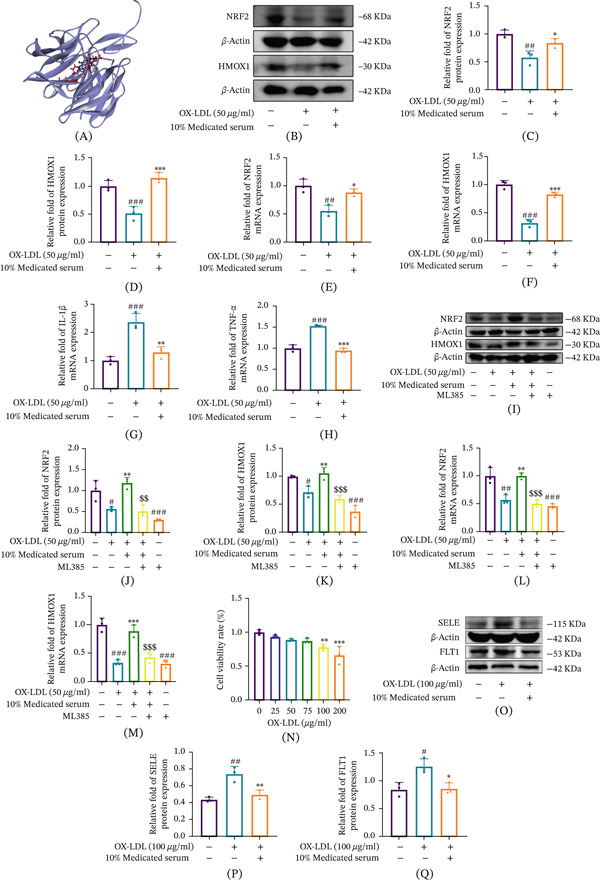
A study on the effects and mechanisms of CSD in models of macrophage foam cell formation and endothelial cell damage. (A) NRF2 and CSD small molecule docking results. (B–D) Protein expression levels of NRF2 and HMOX1.(E–H) mRNA expression levels of NRF2, HMOX1, IL‐1*β* and TNF‐*α*. (I–M) Effects of NRF2 inhibitor ML385 (5 *μ*M, 1 h pre‐treatment) on protein and gene expression of NRF2 and HMOX1. (N) The effects of different concentrations of OX‐LDL on the cell viability of HUVECs. (O–Q) The expression of SELE and FLT1 proteins in HUVECs.

Based on molecular docking results, HMOX1 showed the highest binding affinity with the active component of CSD. Given that HMOX1 expression is regulated by NRF2 and the NRF2/HMOX1 pathway plays a key anti‐inflammatory role in AS, we evaluated the effect of CSD‐mediated serum on this pathway. First, we performed molecular docking between the NRF2 target and small molecules for CSD. The results revealed that DNOP and ellagic acid exhibited high docking scores with NRF2 (Figure 7A). In OX‐LDL‐stimulated macrophages, both mRNA and protein levels of NRF2 and HMOX1 were significantly decreased, while CSD‐mediated serum notably reversed this downregulation (Figure 7B–F). Previous enrichment analysis suggested that the anti‐atherosclerotic effect of CSD may involve suppression of inflammatory responses. Consistent with this, OX‐LDL stimulation markedly increased the expression of TNF‐*α* and IL‐1*β*, which was attenuated by CSD‐mediated serum (Figure 7G,H). To determine whether CSD’s effects depend on the Nrf2/HMOX1 pathway, we used the Nrf2 inhibitor ML385. Pretreatment with ML385 abolished CSD‐induced upregulation of Nrf2 and HMOX1 at both transcriptional and protein levels (Figure 7I–M). In summary, these results indicate the anti‐AS effect of CSD depends on the activity of the NRF2/HMOX1 pathway.

Subsequently, stimulation of HUVECs with varying concentrations of OX‐LDL demonstrated that 100 *μ*g/mL OX‐LDL induced cellular damage in HUVECs. Accordingly, 100 *μ*g/mL OX‐LDL was selected to stimulate HUVECs for the establishment of an endothelial cell injury model (Figure 7N). WB analysis showed that OX‐LDL significantly upregulated the protein expression of FLT1 and SELE, whereas CSD markedly attenuated the OX‐LDL‐induced increase in FLT1 and SELE protein levels (Figure 7O–Q).

## 4. Discussion

This study employed a comprehensive approach combining microarray data, single‐cell sequencing data, and bioinformatics methods to investigate the mechanism of action of the Mongolian herbal medicine CSD against AS. The results revealed that CSD acts on the core targets HMOX1, SELE, FLT1 and CD4, with macrophages and endothelial cells serving as key effector cells. It exerts its therapeutic effects by suppressing inflammatory responses, reducing lipid accumulation and protecting vascular endothelial function. Further studies demonstrated that the anti‐AS effects of CSD depend on the activity of the NRF2/HMOX1 pathway. This elucidated that this pathway serves as the key molecular mechanism through which CSD regulates the pathological progression of AS and exerts its anti‐atherosclerotic effects, thereby providing important theoretical grounds and molecular experimental support for the clinical prevention and treatment of AS using the Mongolian herbal formula CSD.

AS is the main pathological basis of cardiovascular and cerebrovascular diseases, seriously threatening human health [12, 13]. Chronic inflammation and oxidative stress are well‐recognized key factors in AS [14]. AS development is mainly attributed to endothelial dysfunction induced by these responses. Morphologically, dysfunctional endothelial cells show altered structure and increased permeability [15]. Cytoskeletal changes disrupt intercellular junctions, widen intercellular gaps, and damage the endothelial barrier, thereby promoting the entry of low‐density lipoprotein and monocytes into the intima. Damaged endothelial cells secrete adhesion molecules and chemokines [16, 17], which induce monocytes to migrate through endothelial gaps into the intima and differentiate into macrophages. These macrophages then extensively phagocytose oxidized LDL via surface receptors, eventually forming foam cells [18]. The accumulation of foam cells leads to lipid streaks in early AS. The pro‐inflammatory M1 macrophage phenotype promotes AS plaque formation, while the M2 phenotype helps prevent inflammation, reduce plaque size and enhance plaque stability. Based on our previous experiments, we treated RAW264.7 macrophages with 50 *μ*g/mL oxidized LDL to establish an in vitro foam cell model for lipid accumulation and foam cell formation [19]. NRF2 expression was selectively inhibited using 5 *μ*M ML385, which effectively suppresses NRF2 without cytotoxic effects [20].

The study found that CSD acts on different targets through various bioactive compounds and involves multiple signalling pathways to treat AS. Its effects mainly focus on four key targets: HMOX1, SELE, FLT1 and CD4. HMOX1 is primarily located in macrophages and foam cells, and its anti‐inflammatory and antioxidant functions help counteract early AS lesions. HMOX1 catalyses heme cleavage to produce ferrous ions, carbon monoxide and biliverdin [21]. Biliverdin reductase then converts biliverdin to bilirubin. These three products have anti‐inflammatory, antioxidant, anti‐apoptotic and anti‐thrombotic effects. Thus, the anti‐AS effect of HMOX1 is closely linked to its metabolites, and its ability to alleviate oxidative stress protects against AS [22]. The NRF2/HMOX1 pathway plays a critical protective role by suppressing foam cell formation [23]. SELE is a cell adhesion molecule of the selectin family. High SELE expression promotes inflammation. SELE is mainly expressed in endothelial cells and is a key marker of endothelial damage. It mediates leukocyte adhesion to endothelial cells, helping leukocytes migrate to the vascular intima and trigger inflammation, which is an early step in leukocyte recruitment to atherosclerotic plaques [24]. FLT1 is a vascular endothelial growth factor receptor that promotes neovascularization. By acting on FLT1, VEGF promotes endothelial cell proliferation, increases vascular permeability and induces angiogenesis. Intraplaque angiogenesis is a key factor in AS progression and plaque instability. One study [25] showed that increased expression of VEGFR1 and VEGFR2 in macrophages and endothelial cells of human AS plaques is associated with reduced levels of natural antibodies against VEGFR1, which normally suppress inflammatory factors. This suggests that enhanced VEGF and VEGFR signalling promotes angiogenesis and inflammation, leading to plaque instability and possible rupture. These processes contribute to AS formation and progression [26]. CD4 is a glycoprotein on the surface of T cells and serves as a T cell marker. CD4+ T cells are key players in immune responses. ox‐LDL acts as an autoantigen recognized by CD4+ T cells via MHC class II presentation [27]. After antigen recognition, antigen‐presenting cells activate CD4+ T cells, which then differentiate into Th cells and Treg cells. Th1 cells secrete pro‐inflammatory cytokines such as IFN‐*γ* and IL‐2, which promote AS and induce macrophage differentiation into pro‐inflammatory subtypes [28], thereby worsening inflammation and plaque instability.

Molecular docking results showed that the four key targets—HMOX1, CD4, SELE and FLT1—exhibited the highest docking scores with chebulic acid, quercetin, DNOP and kaempferol, respectively. Among these, HMOX1, CD4 and SELE showed the best docking affinity with chebulic acid; while SELE, CD4 and FLT1 showed the best docking results with quercetin. Therefore, chebulic acid and quercetin may be key small molecules for the treatment of AS using CSD. Previous studies have shown [29] that chebulic acid possesses antioxidant and anti‐inflammatory effects, primarily exerting its effects by regulating NRF2‐related signalling pathways. For example, chebulic acid prevents hypoxic damage by activating the Nrf2/ARE pathway, thereby effectively controlling the progression of ischemic stroke. Quercetin plays a multifaceted regulatory role in the progression of AS. By upregulating MST1‐mediated autophagy in RAW264.7 macrophages, quercetin promotes the clearance of foam cells, thereby slowing plaque formation [30]; Quercetin can also regulate the expression of lipid metabolism‐related genes such as PCSK9, CD36, PPAR*γ*, LXR*α* and ABCA1, improving intracellular lipid homeostasis and thereby slowing the progression of AS [31].

This study uses network pharmacology to identify bioactive compounds. Subsequent research could involve the isolation and purification of these compounds to investigate their mechanisms of action at the monomer level. While this study primarily focuses on the regulatory roles of core pathways, future research could be expanded to include upstream and downstream signalling networks to provide a more comprehensive understanding of the overall regulatory mechanisms.

## 5. Conclusion

CSD has demonstrated significant anti‐AS activity. Its therapeutic efficacy has been comprehensively validated using a variety of methods, including multi‐omics studies, molecular docking and cellular experiments, which have elucidated the mechanism of action of CSD in AS. Studies indicate that CSD exerts its anti‐AS effects through the synergistic action of multiple components, targets and pathways. Centred on the regulation of HMOX1, SELE, FLT1 and CD4, and dependent on the NRF2/HMOX1 signalling pathway, it acts on macrophages and endothelial cells to exert anti‐inflammatory effects, reduce lipid deposition and protect the vascular endothelium. This provides a theoretical basis and data support for its subsequent clinical application and modern pharmacological research.

## Author Contributions

Wanlu Sheng and Lina Dai contributed equally to this work.

## Funding

This study was supported by the National Natural Science Foundation of China (61903105); Heilongjiang Provincial Natural Science Foundation (LH2023C063, LH2023C061); Hohhot High‐Level Innovative Talent Project (2023RC‐High‐Level‐14); Inner Mongolia Autonomous Region Public Hospital Research Joint Fund (2024GLLH0248); Research and Development of Peptide Based Anti‐Tumor Drugs Targeting Polo Like Kinases; the Academic Promotion Support Program of Hainan Medical University (XSTS2025036 to W.H.); and Natural Science Foundation of Hainan Province (826MS0160 to W.H.).

## Conflicts of Interest

The authors declare no conflicts of interest.

## Data Availability

The data supporting the findings of this study are available in the Gene Expression Omnibus (GEO) database (https://www.ncbi.nlm.nih.gov/geo/). In addition, these data were derived from publicly available resources. An independent external validation dataset was obtained from Zenodo (https://zenodo.org/):– GSE43292, https://www.ncbi.nlm.nih.gov/geo/query/acc.cgi?acc=GSE43292
– GSE28829, https://www.ncbi.nlm.nih.gov/geo/query/acc.cgi?acc=GSE28829
– GSE159677, https://www.ncbi.nlm.nih.gov/geo/query/acc.cgi?acc=GSE159677
–
https://zenodo.org/records/17454209

## References

[1] Attardo S. , Musumeci O. , Velardo D. , and Toscano A. , Statins Neuromuscular Adverse Effects, International Journal of Molecular Sciences. (2022) 23, no. 15, 10.3390/ijms23158364, 35955495. PMC9369175 35955495

[2] Li S. , Zhao Z. , Aruhan , and Li M. , Mongolian Medicine: From Traditional Practice to Scientific Development, Pharmacological Research. (2023) 197, 106977, 10.1016/j.phrs.2023.106977, 38032295. 38032295

[3] Bai L. and Fu M. , Traditional Mongolian Medicine: Past, Present and Future, Chinese Herbal Medicines. (2022) 14, no. 3, 343–344, 10.1016/j.chmed.2022.06.001, 36118004. 36118004 PMC9476767

[4] Qu S. , Bao J. , Ao W. , Bai L. , and Borjigidai A. , Mongolian Medicine: History, Development and Existing Problems, Chinese Herbal Medicines. (2022) 14, no. 3, 345–355, 10.1016/j.chmed.2022.06.004, 36117997. 36117997 PMC9476632

[5] Li L. and Wang Q. , Water Decoction of Platycladus orientalis Reduces Blood Glucose and Lipids in Mice (In Chinese), Chinese Journal of Pathophysiology. (2017) 33, no. 10, 1814–1818.

[6] Lin J. , Wang X. , Gu M. , Chen Y. , Xu J. , Chau N. V. , Li J. , Ji X. , Chu Q. , Qing L. , and Wu W. , Geniposide Ameliorates Atherosclerosis by Restoring Lipophagy via Suppressing PARP1/PI3K/AKT Signaling Pathway, Phytomedicine : International Journal of Phytotherapy and Phytopharmacology. (2024) 129, 155617, 10.1016/j.phymed.2024.155617, 38614041. 38614041

[7] Liu H. , Chen Y. F. , Li F. , and Zhang H. Y. , Fructus Gardenia (Gardenia jasminoides J. Ellis) Phytochemistry, Pharmacology of Cardiovascular, and Safety With the Perspective of New Drugs Development, Journal of Asian Natural Products Research. (2013) 15, no. 1, 94–110, 10.1080/10286020.2012.723203, 23211013. 23211013

[8] Reddy M. M. , Dhas Devavaram J. , Dhas J. , Adeghate E. , and Starling Emerald B. , Anti-Hyperlipidemic Effect of Methanol Bark Extract of Terminalia chebula in Male Albino Wistar Rats, Pharmaceutical Biology. (2015) 53, no. 8, 1133–1140, 10.3109/13880209.2014.962058, 25625850. 25625850

[9] Li Y. , Zhang L. , Ren P. , Yang Y. , Li S. , Qin X. , Zhang M. , Zhou M. , and Liu W. , Qing-Xue-Xiao-Zhi Formula Attenuates Atherosclerosis by Inhibiting Macrophage Lipid Accumulation and Inflammatory Response via TLR4/MyD88/NF-*κ*B Pathway Regulation, Phytomedicine : International Journal of Phytotherapy and Phytopharmacology. (2021) 93, 153812, 10.1016/j.phymed.2021.153812, 34753029. 34753029

[10] Zhang Z. , Yi P. , Yang J. , Huang J. , Xu P. , Hu M. , Zhang C. , Wang B. , and Peng W. , Integrated Network Pharmacology Analysis and Serum Metabolomics to Reveal the Cognitive Improvement Effect of Bushen Tiansui Formula on Alzheimer′s Disease, Journal of Ethnopharmacology. (2020) 249, 112371, 10.1016/j.jep.2019.112371, 31683034. 31683034

[11] Sadaqat M. , Qasim M. , ul Qamar M. T. , Masoud M. S. , Ashfaq U. A. , Noor F. , Fatima K. , Allemailem K. S. , Alrumaihi F. , and Almatroudi A. , Advanced Network Pharmacology Study Reveals Multi-Pathway and Multi-Gene Regulatory Molecular Mechanism of Bacopa monnieri in Liver Cancer Based on Data Mining, Molecular Modeling, and Microarray Data Analysis, Computers in Biology and Medicine. (2023) 161, 107059, 10.1016/j.compbiomed.2023.107059, 37244150. 37244150

[12] Bułdak Ł. , Cardiovascular Diseases—A Focus on Atherosclerosis, Its Prophylaxis, Complications and Recent Advancements in Therapies, International Journal of Molecular Sciences. (2022) 23, no. 9, 10.3390/ijms23094695, 35563086. PMC9103939 35563086

[13] Fuster J. J. , Maclauchlan S. , and Zuriaga M. A. , Clonal hematopoiesis Associated With TET2 Deficiency Accelerates Atherosclerosis Development in Mice, Science. (2017) 355, no. 6327, 842–847, 10.1126/science.aag1381, 28104796. 28104796 PMC5542057

[14] Fava C. and Montagnana M. , Atherosclerosis Is an Inflammatory Disease Which Lacks a Common Anti-inflammatory Therapy: How Human Genetics Can Help to this Issue. A Narrative Review, Frontiers in Pharmacology. (2018) 9, 10.3389/fphar.2018.00055, 29467655. PMC5808208 29467655

[15] Krüger-Genge A. , Blocki A. , Franke R. P. , and Jung F. , Vascular Endothelial Cell Biology: An Update, International Journal of Molecular Sciences. (2019) 20, no. 18, 10.3390/ijms20184411, 31500313. PMC6769656 31500313

[16] Ridker P. M. and Rane M. , Interleukin-6 Signaling and Anti-Interleukin-6 Therapeutics in Cardiovascular Disease, Circulation Research. (2021) 128, no. 11, 1728–1746, 10.1161/CIRCRESAHA.121.319077, 33998272. 33998272

[17] Georgakis M. K. , Van Der Laan S. W. , Asare Y. , Mekke J. M. , Haitjema S. , Schoneveld A. H. , de Jager S. C. , Nurmohamed N. S. , Kroon J. , Stroes E. S. , and de Kleijn D. P. , Monocyte-Chemoattractant Protein-1 Levels in Human Atherosclerotic Lesions Associate With Plaque Vulnerability, Arteriosclerosis, Thrombosis, and Vascular Biology. (2021) 41, no. 6, 2038–2048, 10.1161/ATVBAHA.121.316091, 33827260. 33827260

[18] Gong S. , Li Y. , Yan K. , Shi Z. , Leng J. , Bao Y. , and Ning K. , The Crosstalk Between Endothelial Cells, Smooth Muscle Cells, and Macrophages in Atherosclerosis, International Journal of Molecular Sciences. (2025) 26, no. 4, 10.3390/ijms26041457, 40003923. PMC11855868 40003923

[19] Yang X. , Ma L. , Zhang J. , Chen L. , Zou Z. , Shen D. , He H. , Zhang L. , Chen J. , Yuan Z. , Qin X. , and Yu C. , Hypofucosylation of Unc5b Regulated by Fut8 Enhances Macrophage Emigration and Prevents Atherosclerosis, Cell & Bioscience. (2023) 13, no. 1, 10.1186/s13578-023-00959-y, 36670464. PMC9854080 36670464

[20] Tao Y. , Zhao Q. , Lu C. , Yong W. , Xu M. , Wang Z. , and Leng X. , Melatonin Suppresses Atherosclerosis by Ferroptosis Inhibition via Activating NRF2 Pathway, FASEB Journal. (2024) 38, no. 10, e23678, 10.1096/fj.202400427RR, 38780199. 38780199

[21] Ayer A. , Zarjou A. , Agarwal A. , and Stocker R. , Heme Oxygenases in Cardiovascular Health and Disease, Physiological Reviews. (2016) 96, no. 4, 1449–1508, 10.1152/physrev.00003.2016, 27604527. 27604527 PMC5504454

[22] Araujo J. A. , Zhang M. , and Yin F. , Heme Oxygenase-1, Oxidation, Inflammation, and Atherosclerosis, Frontiers in Pharmacology. (2012) 3, 119, 10.3389/fphar.2012.00119, 22833723. 22833723 PMC3400084

[23] Zhu H. , Jia Z. , Zhang L. , Yamamoto M. , Misra H. P. , Trush M. A. , and Li Y. , Antioxidants and Phase 2 Enzymes in Macrophages: Regulation by Nrf2 Signaling and Protection Against Oxidative and Electrophilic Stress, Experimental Biology and Medicine. (2008) 233, no. 4, 463–474, 10.3181/0711-RM-304, 18367636. 18367636

[24] Ma S. , Tian X. Y. , Zhang Y. , Mu C. , Shen H. , Bismuth J. , Pownall H. J. , Huang Y. , and Wong W. T. , E-Selectin-Targeting Delivery of microRNAs by Microparticles Ameliorates Endothelial Inflammation and Atherosclerosis, Scientific Reports. (2016) 6, no. 1, 22910, 10.1038/srep22910, 26956647. 26956647 PMC4783714

[25] Inoue M. , Itoh H. , Ueda M. , Naruko T. , Kojima A. , Komatsu R. , Doi K. , Ogawa Y. , Tamura N. , Takaya K. , Igaki T. , Yamashita J. , Chun T. H. , Masatsugu K. , Becker A. E. , and Nakao K. , Vascular Endothelial Growth Factor (VEGF) Expression in Human Coronary Atherosclerotic Lesions: Possible Pathophysiological Significance of VEGF in Progression of Atherosclerosis, Circulation. (1998) 98, no. 20, 2108–2116, 10.1161/01.cir.98.20.2108, 9815864. 9815864

[26] Amare Y. E. , Vuerich R. , and Zacchigna S. , VEGFR1 as a Target for Cardiovascular Gene Therapy, Journal of Cardiovascular Translational Research. (2025) 18, no. 6, 1485–1502, 10.1007/s12265-025-10672-5, 40839179. 40839179

[27] Colantonio L. D. , Bittner V. , Reynolds K. , Levitan E. B. , Rosenson R. S. , Banach M. , Kent S. T. , Derose S. F. , Zhou H. , Safford M. M. , and Muntner P. , Association of Serum Lipids and Coronary Heart Disease in Contemporary Observational Studies, Circulation. (2016) 133, no. 3, 256–264, 10.1161/CIRCULATIONAHA.115.011646, 26659948. 26659948 PMC4718875

[28] Orecchioni M. , Ghosheh Y. , Pramod A. B. , and Ley K. , Corrigendum: Macrophage Polarization: Different Gene Signatures in M1(LPS+) vs. Classically and M2(LPS-) vs. Alternatively Activated Macrophages, Frontiers in Immunology. (2020) 11, no. 2019, 10.3389/fimmu.2020.00234, 32161587. PMC7053374 32161587

[29] Zhou R. , Lin K. , Leng C. , Zhou M. , Zhang J. , Li Y. , Liu Y. , Ye X. , Xu X. , Sun B. , Shu X. , and Liu W. , Chebulic Acid Prevents Hypoxia Insult via Nrf2/ARE Pathway in Ischemic Stroke, Nutrients. (2022) 14, no. 24, 10.3390/nu14245390, 36558549. PMC9781341 36558549

[30] Cao H. , Jia Q. , Yan L. , Chen C. , Xing S. , and Shen D. , Quercetin Suppresses the Progression of Atherosclerosis by Regulating MST1-Mediated Autophagy in ox-LDL-Induced RAW264.7 Macrophage Foam Cells, International Journal of Molecular Sciences. (2019) 20, no. 23, 10.3390/ijms20236093, 31816893. PMC6928812 31816893

[31] Jia Q. , Cao H. , Shen D. , Li S. , Yan L. , Chen C. , Xing S. , and Dou F. , Quercetin Protects Against Atherosclerosis by Regulating the Expression of PCSK9, CD36, PPAR*γ*, LXR*α* and ABCA1, International Journal of Molecular Medicine. (2019) 44, no. 3, 893–902, 10.3892/ijmm.2019.4263, 31524223. 31524223 PMC6658003

